# A risk prediction nomogram for in-stent restenosis in patients with coronary heart disease: first exploratory analysis based on the substrate materials of drug-eluting stents

**DOI:** 10.3389/fcvm.2025.1549212

**Published:** 2026-01-12

**Authors:** Zheng Zhao, Kai Li, Kai Tan, Rui Zhang, Jiawei Tian, Rong Li, Shaohua Li, Shaoyan Liu, Fei Yu, Hui Xin

**Affiliations:** 1 Department of Cardiology, The Affiliated Hospital of Qingdao University, Qingdao University, Qingdao, China; 2 School of Basic Medicine, Qingdao Medical College, Qingdao University, Qingdao, China; 3 Department of Cardiology I, Qingdao Central Hospital, University of Health and Rehabilitation Sciences, Qingdao, China; 4 Department of Emergency Internal Medicine, The Affiliated Hospital of Qingdao University, Qingdao University, Qingdao, China

**Keywords:** nomogram, risk factors, in-stent restenosis (ISR), percutaneous coronary intervention (PCI), drug-eluting stent (DES)

## Abstract

**Objective:**

We aimed to develop and validate a predictive nomogram for identifying the risk factors of in-stent restenosis (ISR). In addition, for the first time, we quantified the clinical effect of the substrate materials of DES.

**Methods:**

We retrospectively enrolled 402 patients with coronary heart disease (CHD) who underwent initial percutaneous coronary intervention (PCI) at the Affiliated Hospital of Qingdao University between January 1, 2012, and June 1, 2022. LASSO regression and logistic regression analyses were conducted to identify the independent risk factors of ISR. A predictive nomogram was subsequently developed. Model performance was assessed using the area under the receiver operating characteristic curve (AUC), decision curve analysis (DCA), clinical impact curve (CIC), and calibration curves. Furthermore, nested modeling was conducted to evaluate the incremental predictive value of the substrate materials of DES.

**Results:**

BMI, SBP, LVDD, number of target vessels, mean diameter of stent and substrate materials of DES were identified as independent predictors of ISR. A predictive nomogram model was successfully developed, exhibiting good performance in the training set (AUC = 0.734, 95% CI: 0.676–0.792; Brier score = 0.193, 95% CI: 0.173–0.213; calibration slope = 1.000, 95% CI: 0.706–1.359; Hosmer-Lemeshow *χ*^2^ = 8.087, *P* = 0.088). In addition, the nomogram model maintained stable performance in the validation set (AUC = 0.707, 95% CI: 0.562–0.837; Brier score = 0.207, 95% CI: 0.161–0.258; calibration slope = 0.842, 95% CI: 0.229–1.991; Hosmer-Lemeshow *χ*^2^ = 2.641, *P* = 0.620). The base model, including the substrate materials of DES in the nested analysis, was well-calibrated (*χ*^2^ = 8.087, *P* = 0.088; Brier score = 0.1929). However, the removal of this predictor significantly deteriorated calibration(*χ*^2^ = 14.0, *P* = 0.007; Brier score = 0.1962, Δ =  + 0.0033) and worsened reclassification metrics (continuous NRI = −0.2549, 95% CI: −0.4635 to −0.0481, *P* = 0.021; IDI = −0.0134, 95% CI: −0.0507 to −0.003, *P* = 0.033).

**Conclusions:**

BMI, SBP, LVDD, number of target vessels, mean diameter of stent, and substrate materials of DES are independent predictors of ISR. The nomogram model exhibited good predictive value for ISR. This is the first study demonstrating the significance of substrate material selection for assessing the risk of ISR in patients. Future validation through prospective studies or larger sample sizes is still needed.

## 1 Introduction

An increasing number of countries are suffering from aging and its complications, such as coronary artery disease (CAD). CAD, comprising acute coronary syndrome (ACS) and stable angina pectoris (SAP), has emerged as a leading cause of mortality worldwide (1, 2). Percutaneous coronary intervention (PCI) is now the primary revascularization strategy for CAD (3). This procedure effectively dilates stenosed or occluded coronary arteries and alleviates clinical symptoms, offering high safety and minimal surgical trauma (4, 5). Approximately 60% of patients with ACS are treated with PCI, and this proportion is increasing annually (6, 7).

The long-term success of PCI is significantly limited by in-stent restenosis (ISR) (8, 9). ISR is defined as stenosis exceeding 50% of the previously stent-implanted segment or its adjacent segment (10), characterized by delayed recurrence of ischemic symptoms after a successful PCI (11). While bare metal stents (BMS) prevent elastic recoil and constrictive remodeling (12), they can increase the risk of ISR (up to 30%) (13). Drug-eluting stents (DES) reduce ISR rates to 5%–10% (14). Approximately 50% of ISR cases present with unstable angina (UA), with 18.7% progressing to non-ST-segment elevation myocardial infarction (NSTEMI) and 8.5% progressing to ST-segment elevation myocardial infarction (STEMI) (15). Patients with DES-related ISR experience UA more frequently than those with *de novo* stenosis (61% vs. 45%, *p* < 0.001). Furthermore, 17% of patients with DES-related ISR experience major adverse cardiovascular events (*p* < 0.001) (16). Therefore, ISR remains a critical concern after PCI (17).

The factors associated with ISR have attracted much attention, including patient's demographic factors (age, gender, history of hypertension, history of hyperlipidemia, history of diabetes, history of smoking, history of drinking, left ventricular function, STEMI, history of bypass surgery, history of congestive heart failure, history of chronic kidney disease, history of chronic obstructive pulmonary disease, family history of coronary heart disease, withdrawal of aspirin, and use of conventional dose statins), biological factors (drug resistance, hs-CRP, angiotensin-converting enzyme, serum matrix metalloproteinases, genetics, GS score, hemoglobin level, reticulocyte count, monocyte count, neutrophil/lymphocyte ratio, platelet/lymphocyte ratio, red blood cell distribution width, average platelet volume, and platelet distribution width), pathological factors (lesion location, lesion length, bifurcation lesion, tandem lesion, small vessel lesion, inflammation, allergic reaction, new atherosclerosis, calcified nodule, plaque prolapse or protrusion, edge peeling, number of stents, type of stents, and duration of stent implantation) and mechanical factors (insufficient stent expansion, stent fracture, overexpansion of undersized stents, uneven drug deposition, polymer damage, stent pillar misalignment, residual stenosis, and stent gap) (18–21). Inconsistent findings of previous studies regarding these predictive factors are due to differences in study design, sample size, methodology, and the measured variables. It is crucial to note that there is no statistical analysis on the substrate materials of DES as a risk factor, necessitating further studies.

This study aimed to analyze preoperative demographic, clinical, laboratory, lesion-specific, and procedural characteristics in patients suffering from ISR. Specifically, we evaluated the predictive ability of the substrate materials of DES as a risk factor. This analysis provides novel insights for developing predictive nomograms. Such tools can help clinicians identify patients at a high risk of ISR earlier and optimize treatment strategies, finally improving patients' outcomes.

## 2 Methods

### 2.1 Study population

**Figure 1:**
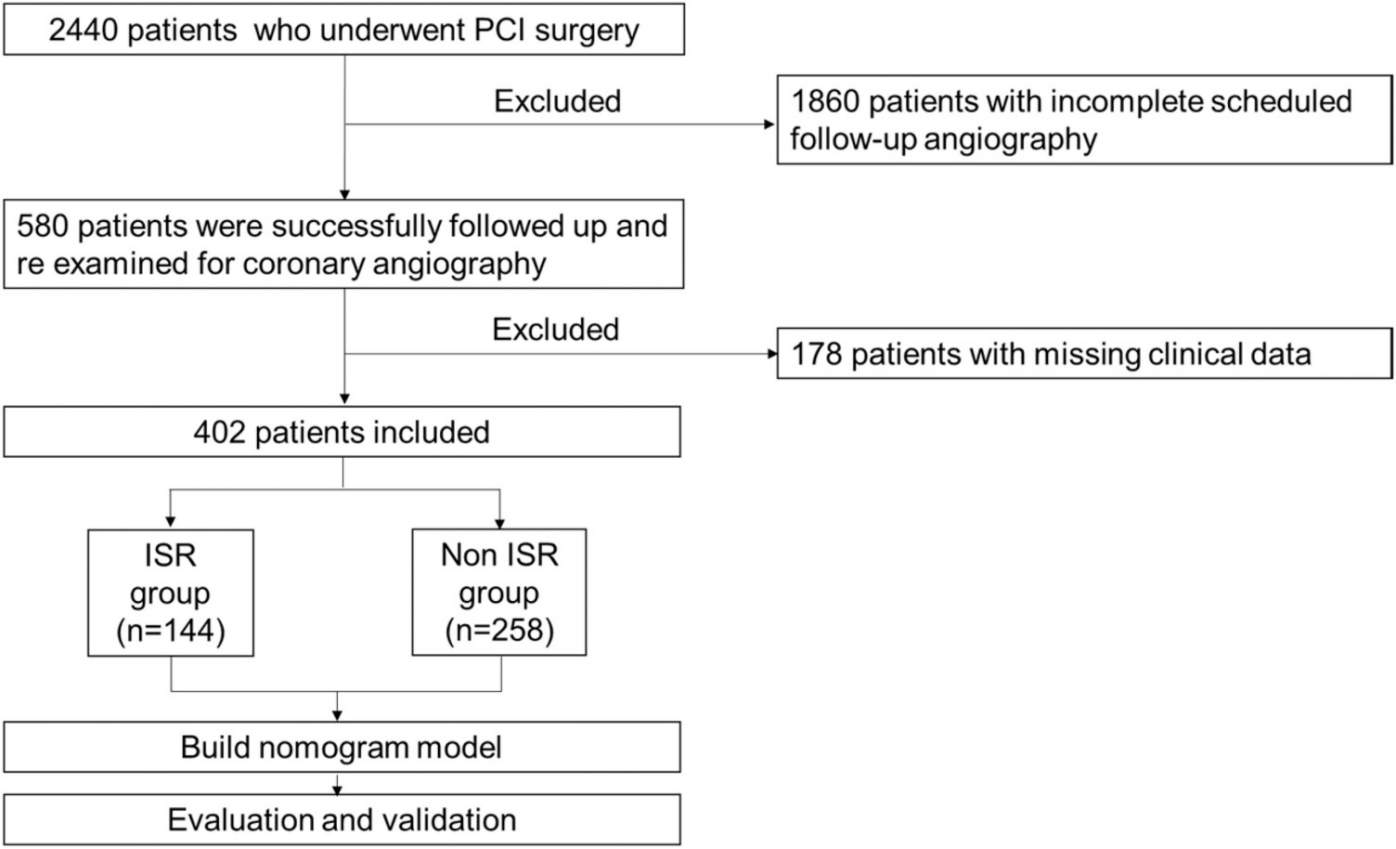
Study flowchart.

This study initially screened 2,440 patients diagnosed with coronary heart disease (CHD) who underwent their first percutaneous coronary intervention (PCI) at the Affiliated Hospital of Qingdao University between January 1, 2012, and June 1, 2022. The final cohort of 402 patients was derived by sequentially applying the inclusion and exclusion criteria (detailed in Figure 1), whereby patients with missing key data (e.g., laboratory results) or who met any exclusion criterion were omitted, consistent with a complete-case analysis approach. All included patients underwent follow-up coronary angiography (CAG) with a median follow-up time of 18.86 months and received standard post-operative statin and antiplatelet therapy. This study was approved by the Institutional Review Committee of the Affiliated Hospital of Qingdao University (Approval No. QYFY WZLL 28549) and adhered to relevant ethical guidelines and regulations. The inclusion criteria were as follows: (i) acute coronary syndrome; (ii) first-time PCI at this hospital; (iii) PCI procedure conducted in accordance with the Chinese guidelines for PCI (2016); (iv) incidence of in-stent restenosis (ISR) confirmed by follow-up coronary angiography; (v) availability of complete demographic, laboratory, and imaging data from the hospital information system. The exclusion criteria were as follows: (i) underwent PCI at another hospital during follow-up; (ii) underwent coronary artery bypass grafting during the follow-up period; (iii) history of heart failure, myocarditis, pericarditis, cardiomyopathy, congenital heart disease, or other organic heart diseases; (iv) severe liver or kidney dysfunction; (v) comorbidities including immune diseases, infectious diseases, tumor, hematologic diseases, or severe trauma.

### 2.2 Data acquisition

All clinical data were obtained from the information system of the Affiliated Hospital of Qingdao University. According to clinical characteristics and previous studies, 36 candidate variables were selected for further processing, mainly including demographic data (age, gender, smoking history, drinking history, body mass index (BMI), heart rate (HR), systolic blood pressure (SBP), diastolic blood pressure (DBP), and family history of coronary heart diseases), past medical history (history of hypertension, history of diabetes, and history of dyslipidemia), laboratory indicators [fasting blood glucose (FBG), cholesterol (CHOL), triglycerides (TG), high-density lipoprotein (HDL), low-density lipoprotein (LDL), glomerular filtration rate (GFR), and fibrinogen (FIB)], echocardiographic features [left ventricular ejection fraction (LVEF) and left ventricular end-diastolic diameter (LVDD)], and the characteristics of the first and second coronary angiography (onset form, lesion vessel, location of the stenosis, diffuse lesion, degree of stenosis, number of stents, stent diameter, stent length, and substrate material of the stent). These data were obtained from perioperative and follow-up examinations. All patients underwent standardized PCI strategies. Patients were usually treated with repeated coronary angiography after PCI to confirm the incidence of ISR. Early coronary angiography was considered for patients experiencing chest tightness and pain postoperatively. Two experienced cardiologists from our hospital conducted CAG and evaluated the results of imaging. Quantitative analysis of coronary arteries was conducted to visually quantify the degree of coronary artery stenosis. Based on the results of quantitative coronary angiography (QCA) during follow-up CAG, all patients were divided into the ISR group and the non-ISR group.

### 2.3 Statistical analysis

Statistical analyses were conducted using R software (version 4.3) and SPSS (version 27.0). Continuous variables are expressed as mean ± standard deviation or median (interquartile range, IQR). They were compared using independent samples *t*-tests, Mann–Whitney *U* tests, ANOVA, or Kruskal–Wallis *H* tests as appropriate. Categorical variables were presented as counts (percentages) and were compared using the Chi-square test or Fisher's exact test. A two-tailed *p*-value < 0.05 was deemed statistically significant.

Variables with *p* ≥ 0.05 in univariate analysis were excluded, and between-variable correlations were assessed. Subsequently, the Least Absolute Shrinkage and Selection Operator (LASSO) regression was employed to identify the optimal set of predictive variables, addressing potential overfitting. The optimal penalty parameter (*λ*) was determined via 10-fold cross-validation (detailed criteria are provided in supplementary methods). Regression coefficients from the LASSO model were used to calculate odds ratios (OR). The robustness of the LASSO-selected variables was assessed using bootstrap resampling (*n* = 100). Variables selected in ≥80% of bootstrap samples were considered highly stable.

The dataset (*n* = 402) was randomly split into the training set (*n* = 335, ∼83.3%) for model development and the independent validation set (*n* = 67, ∼16.7%) for external validation. Baseline characteristics of the two sets were comparable (all *p* > 0.05), confirming successful randomization.

Univariate logistic regression was conducted for each variable. Variables with significant association (*p* < 0.05) in univariate analysis were used in multivariable logistic regression analysis. A backward selection procedure was adopted, retaining variables with *p* < 0.05 in the final model. Multicollinearity was assessed using the variance inflation factor (VIF), with VIF < 5 deemed acceptable.

A nomogram was constructed based on the final multivariable logistic regression model to visualize ISR risk prediction.

Concomitantly, we used bootstrap resampling (*n* = 1,000) to correct for optimism. Model performance was assessed based on the area under the receiver operating characteristic curve (AUC), Brier score, and calibration (Hosmer-Lemeshow test and calibration slope). The performance of the model was then rigorously evaluated in the validation set.

A nested model comparison was conducted to specifically evaluate the predictive contribution of the substrate material of DES. First, we compared a baseline model comprising substrate material with a reduced model excluding it. Multiple dimensions were compared, including model fit (AIC, likelihood ratio test), discrimination (AUC difference tested with DeLong test), calibration (Hosmer-Lemeshow, Brier score), reclassification (Net reclassification improvement-NRI, integrated discrimination improvement-IDI), clinical utility (decision curve analysis-DCA), robustness (cross-validated AUC), and coefficient stability.

## 3 Results

### 3.1 Baseline characteristics

This study included 402 patients undergoing PCI. Among them, 144 (35.8%) developed ISR over a mean follow-up of 36.68 months. They were divided into the non-ISR (N-ISR, *n* = 258) group and the ISR (*n* = 144) group. Thirty-two variables, including clinical characteristics, laboratory/examination findings, lesion features, and procedural details, were analyzed as potential risk factors. The following baseline characteristics revealed no significant differences between the two groups (*P* > 0.05): age, gender, smoking/drinking history, HR, DBP, histories of hypertension, diabetes, dyslipidemia or coronary heart disease, FBG, CHOL, TG, HDL, LDL, GFR, form of onset, number of target lesions, presence of diffuse lesions, degree of stenosis, number of implanted stents, minimum stent diameter, and presence of disease in the left main artery, left anterior descending artery (LAD), left circumflex artery (LCX), right coronary artery (RCA), diagonal branch, obtuse marginal branch (OM), posterior descending artery (PDA), posterolateral branch (PLB), and right ventricular posterior branch (RVPB). In contrast, a significant difference was observed for BMI, SBP, FIB, LVEF, LVDD, the number of target vessels, the mean diameter of stent, the mean length of stent, intermediate branch disease, and the substrate material of DES (*P* < 0.05). Variables showing a significant difference between groups are shown in Table 1. Full baseline characteristics are presented in Supplementary Table S1.

**Table 1:** Baseline characteristics of the study population.

Characteristic	Non ISR group	ISR group	*P*-value
	(*n* = 258)	(*n* = 144)	
BMI, (kg/m^2^)	25.655 (23.66,27.7)	27.36 (24.65,29.905)	<0.001
SBP, (mmHg)	130.5 (120,147)	136 (120.25,150)	0.038
FIB, (mg/Dl)	2.9 (2.425,3.47)	3.03 (2.505,3.858)	0.03
LVEF, (%)	62 (59,64.25)	60.5 (52.25,64)	0.007
LVDD, (mm)	4.6 (4.4,4.9)	4.9 (4.6,5.2)	<0.001
Number of target vessels	3 (2,4)	2 (2,3)	0.017
Mean diameter of stent, (mm)	2.88 (2.63,3.08)	2.75 (2.56,3)	0.029
Mean length of stent, (mm)	22 (18,26.543)	23 (18.25,28)	0.024
Target vessel, (%)			
Intermediate branch	6 (2.3)	10 (6.9)	0.023
Substrate material of DES, (%)			0.046
316L-SS	86 (33.3)	65 (45.1)	
Co-Cr	118 (45.7)	46 (31.9)	
Pt-Cr	28 (10.9)	16 (11.1)	
Co-Ni	26 (10.1)	17 (11.8)	

### 3.2 Correlations between variables

**Figure 2:**
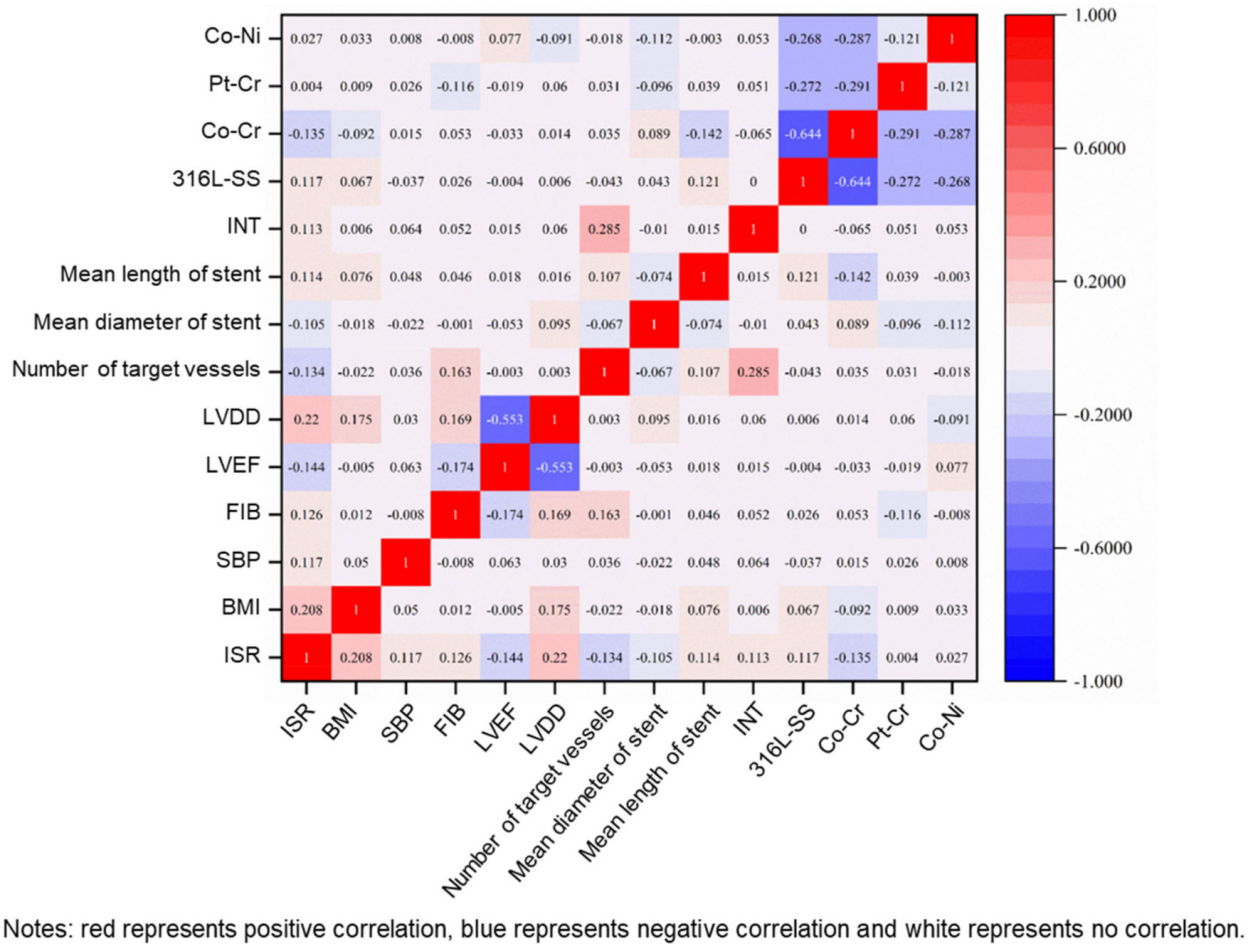
Correlation matrix of variables.

Baseline variables showing significant differences were subjected to multivariate correlation analysis (Figure 2). Significant correlations were observed between ISR and specific variables. Specifically, BMI, SBP, FIB, LVDD, the mean length of stent, INT, 316L-SS, Pt-Cr, and Co-Ni were positively correlated with ISR. Conversely, LVEF, the number of target vessels, the mean diameter of stent, and Co-Cr revealed negative correlations with ISR.

### 3.3 LASSO regression analysis

**Figure 3:**
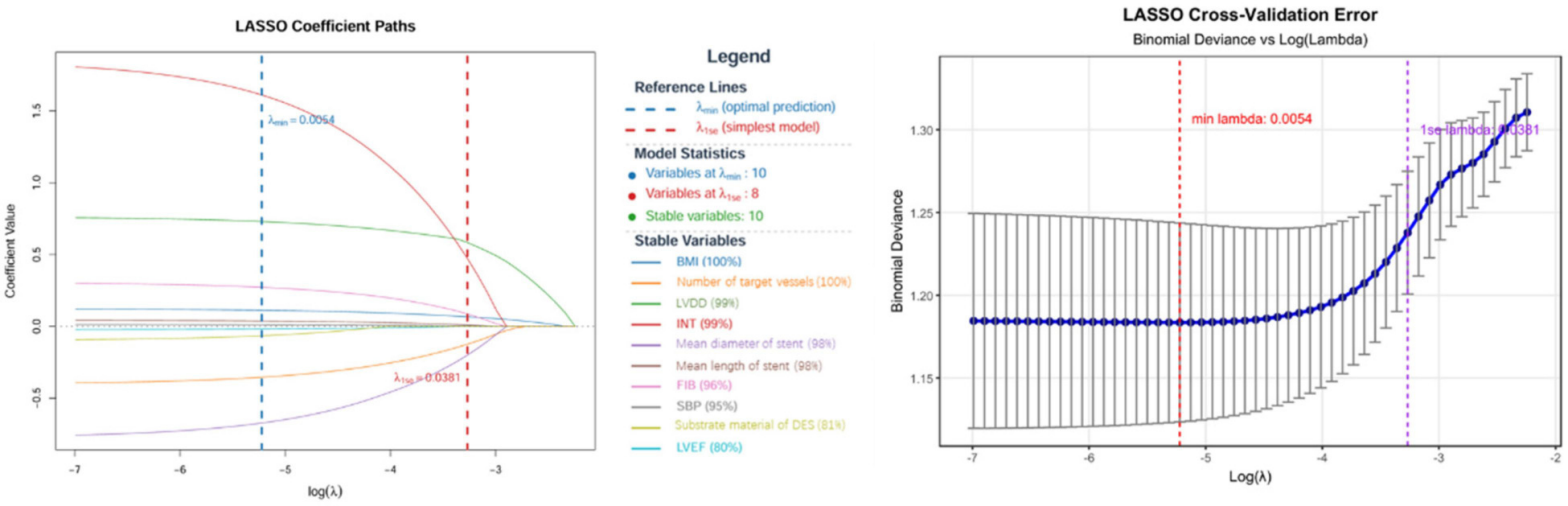
LASSO regression analysis for variable selection.

All 10 predictors retained significance in LASSO regression analysis at lambda.min (*λ* = 0.0122), with INT exhibiting the strongest association (*β* = 1.611, OR = 5.008), followed by LVDD (*β* = 0.730, OR = 2.074) and FIB (*β* = 0.272, OR = 1.313) (Table 2). Bootstrap validation (100 iterations) confirmed high stability (Figure 3). All variables exceeded the 80% selection threshold, and all coefficient standard deviations were <0.15. BMI (100%), the number of target vessels (100%), INT (99%), LVDD (99%), the mean diameter of stent (98%), the mean length of stent (98%), FIB (96%), and SBP (95%) showed exceptional stability (≥95%). The substrate material of DES (81%) and LVEF (80%) exhibited high stability. Although model simplification using lambda.1se (*λ* = 0.0358) reduced the number of predictors to 8, the results showed less accuracy; therefore, it was excluded.

**Table 2:** Stability assessment of LASSO variable selection.

Predictor	Coef	OR	Selection Freq	Selection Rate	Mean Coef	SD Coef
BMI	0.112	1.119	100.000	100.000	0.117	0.039
Number of target vessels	−0.354	0.702	100.000	100.000	−0.365	0.085
INT	1.611	5.008	99.000	99.000	1.645	0.588
LVDD	0.730	2.074	99.000	99.000	0.765	0.338
Mean diameter of stent	−0.673	0.510	98.000	98.000	−0.683	0.333
Mean length of stent	0.037	1.038	98.000	98.000	0.038	0.018
FIB	0.272	1.313	96.000	96.000	0.275	0.130
SBP	0.011	1.011	95.000	95.000	0.010	0.006
Substrate material of DES	−0.065	0.937	81.000	81.000	−0.099	0.121

### 3.4 Univariate and multivariate logistic regression analysis

Univariate analysis identified 8 significant predictors of ISR, including BMI (OR = 1.158, 95% CI = 1.078–1.245, *P* < 0.001), SBP (OR = 1.012, 95% CI = 1.000–1.023, *P* = 0.043), FIB (OR = 1.313, 95% CI = 1.001–1.721, *P* = 0.049), LVDD (OR = 3.282, 95% CI = 1.878–5.735, *P* < 0.001), LVEF (OR = 0.955, 95% CI = 0.925–0.987, *P* = 0.006), the number of target vessels (OR = 0.799, 95% CI = 0.671–0.952, *P* = 0.012), the mean diameter of stent (OR = 0.546, 95% CI = 0.303–0.986, *P* = 0.045), and the substrate material of DES (OR = 0.532, 95% CI = 0.321–0.885, *P* = 0.015). In contrast, INT (*P* = 0.184) and the mean length of stent (*P* = 0.182) showed no statistical significance (Table 3).

**Table 3:** Univariate logistic regression analysis of candidate predictors.

Predictors	OR	95% CI	*P*-value
BMI	1.158	(1.078–1.245)	0.000
SBP	1.012	(1.000–1.023)	0.043
FIB	1.313	(1.001–1.721)	0.049
LVDD	3.282	(1.878–5.735)	0.000
LVEF	0.955	(0.925–0.987)	0.006
Number of target vessels	0.799	(0.671–0.952)	0.012
Mean diameter of stent	0.546	(0.303–0.986)	0.045
Mean length of stent	1.027	(0.988–1.067)	0.182
INT	2.129	(0.699–6.486)	0.184
Substrate material of DES (316LSS)	1.000	(Reference)	0.109
CoCr	0.532	(0.321–0.885)	0.015
PtCr	0.667	(0.296–1.499)	0.327
CoNi	0.713	(0.339–1.499)	0.372

Multivariate analysis identified 6 independent predictors, including BMI (OR = 1.133, 95% CI = 1.049–1.224, *P* = 0.002), SBP (OR = 1.013, 95% CI = 0.902–1.587, *P* = 0.038), LVDD (OR = 2.525, 95% CI = 2.455–2.613, *P* = 0.011), the number of target vessels (OR = 0.762, 95% CI = 0.631–0.921, *P* = 0.005), the mean diameter of stent (OR = 0.445, 95% CI = 0.227–0.870, *P* = 0.018), and the substrate material of DES (OR = 0.571, 95% CI = 0.329–0.992, *P* = 0.047). Conversely, FIB (*P* = 0.094) and LVEF (*P* = 0.449) did not show statistical significance in the multivariate model (Table 4).

**Table 4:** Multivariable logistic regression analysis of candidate predictors.

Predictors	OR	95% CI	*P*-value
BMI	1.133	(1.049–1.224)	0.002
SBP	1.013	(0.902–1.587)	0.038
FIB	1.306	(0.955–1.784)	0.094
LVDD	2.525	(2.455–2.613)	0.011
LVEF	0.983	(0.941–1.027)	0.449
Number of target vessels	0.762	(0.631–0.921)	0.005
Mean diameter of stent	0.445	(0.227–0.870)	0.018
Substrate material of DES (316LSS)	1.000	(Reference)	0.253
CoCr	0.571	(0.329–0.992)	0.047
PtCr	0.702	(0.290–1.695)	0.431
CoNi	0.671	(0.296–1.525)	0.341

### 3.5 Diagnosis of collinearity

**Figure 4:**
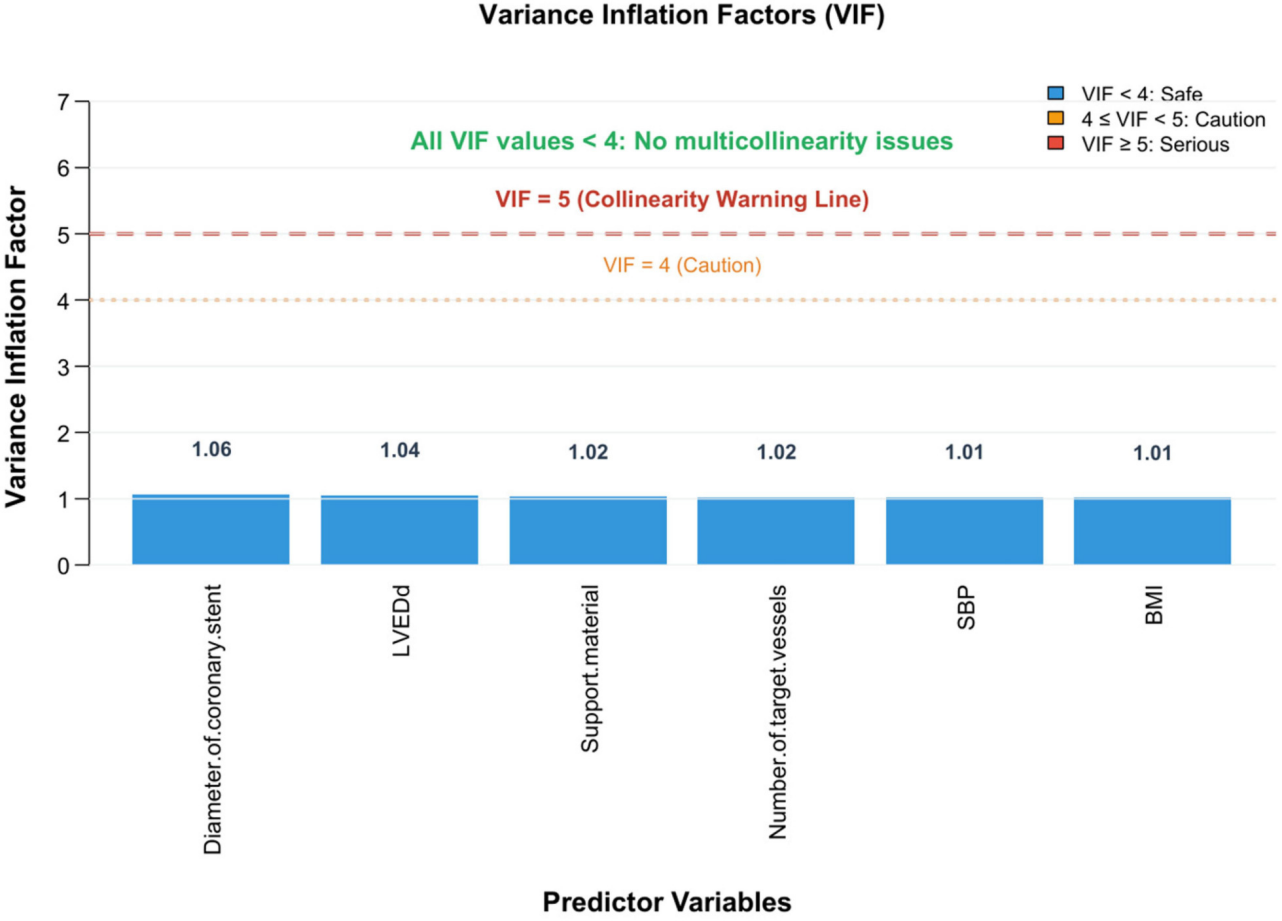
Collinearity diagnosis of predictors.

Variance inflation factor (VIF) assessment confirmed minimal multicollinearity for the independent predictors: the mean diameter of stent (VIF = 1.060), LVDD (VIF = 1.040), the substrate material of DES (VIF = 1.022), the number of target vessels (VIF = 1.020), SBP (VIF = 1.011), and BMI (VIF = 1.009). The six-variable model exhibited a mean VIF of 1.025 ± 0.019 (SD), suggesting negligible inter-variable linear associations (Figure 4).

### 3.6 Construction of the nomogram model

**Figure 5:**
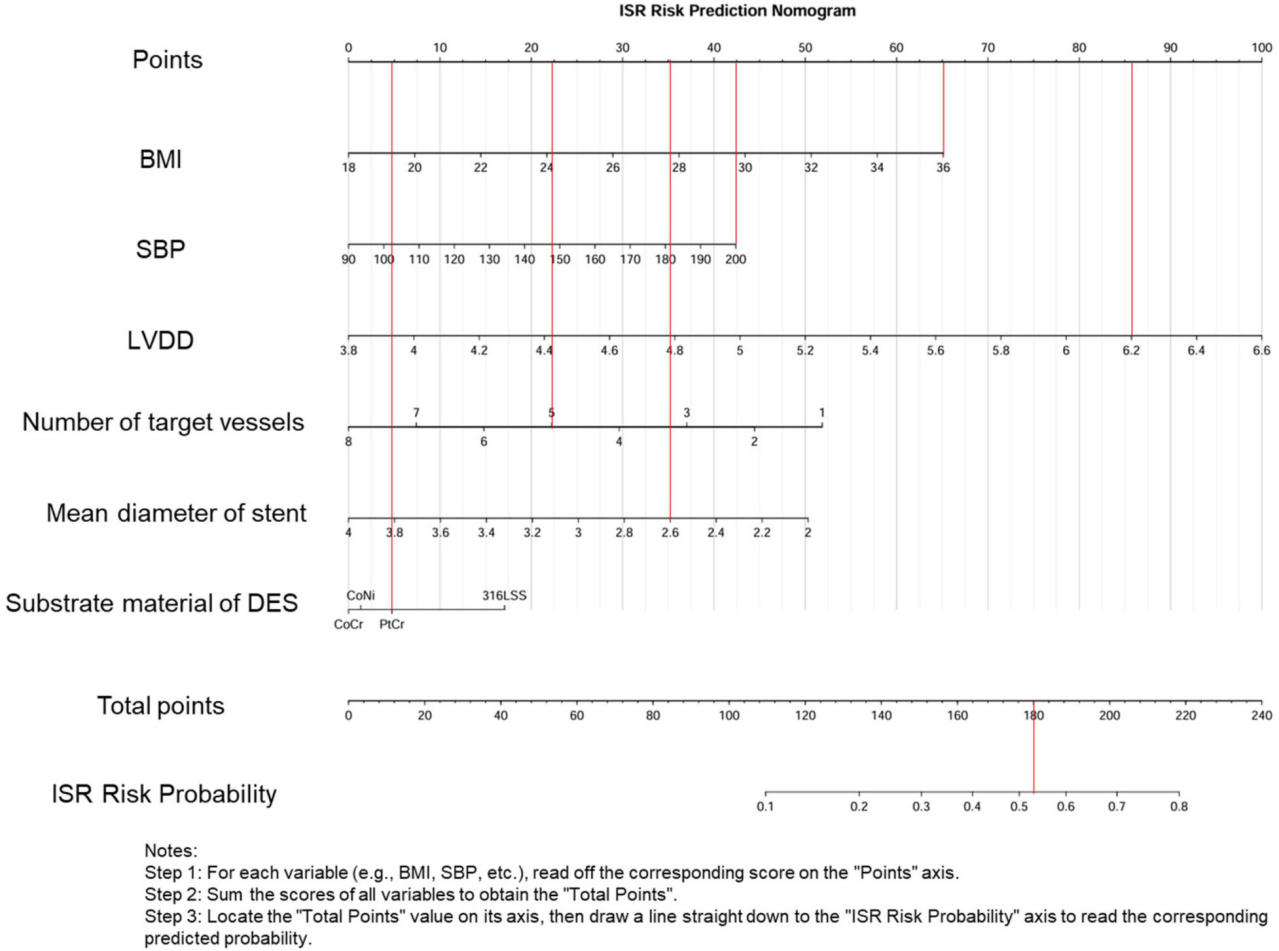
Nomogram for predicting ISR.

**Figure 6:**
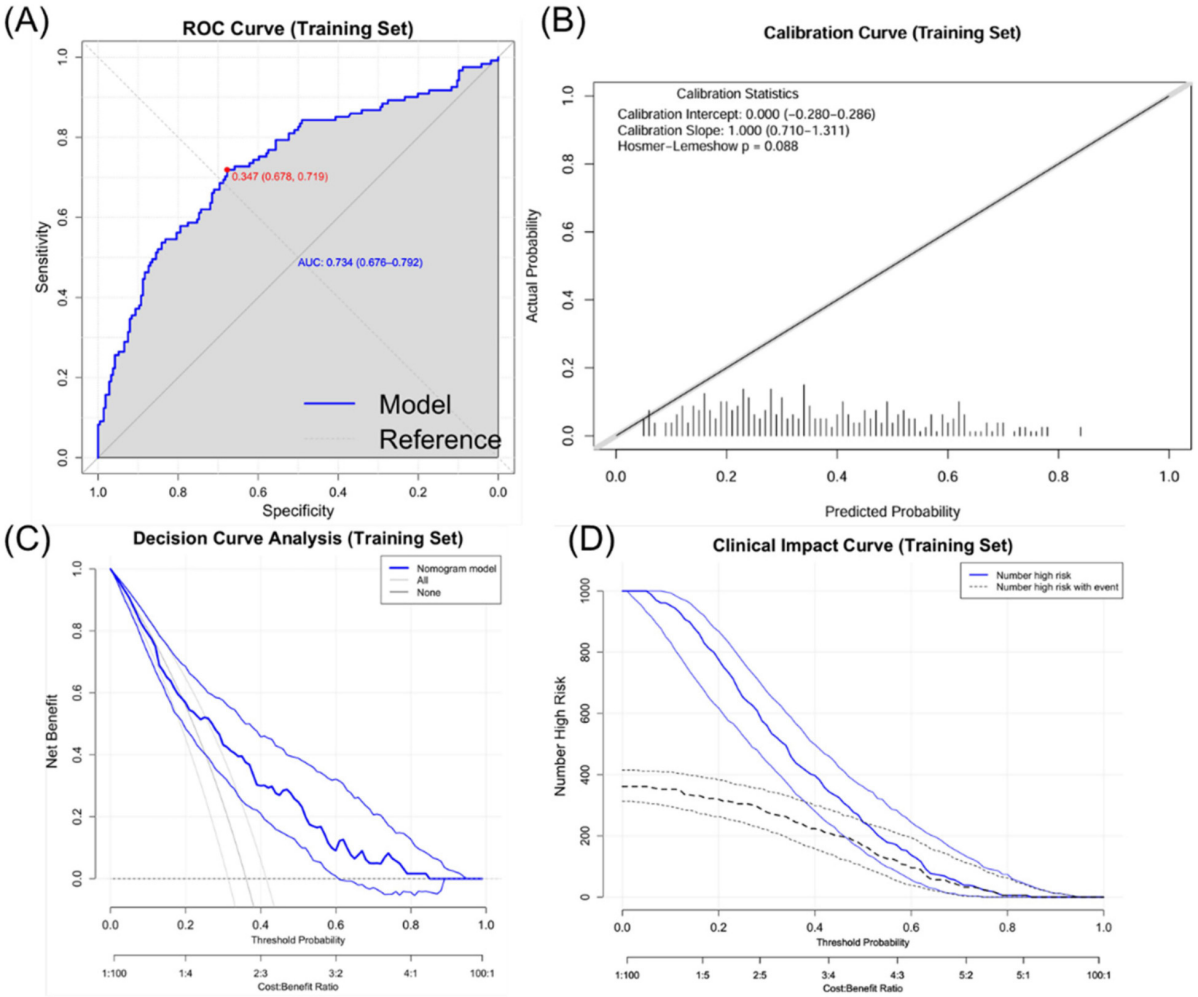
Evaluation of the ISR prediction nomogram (training set). **(A)** Receiver operating characteristic (ROC) curve. **(B)** Calibration curve. **(C)** Clinical impact curve. **(D)** Decision curve analysis.

**Figure 7:**
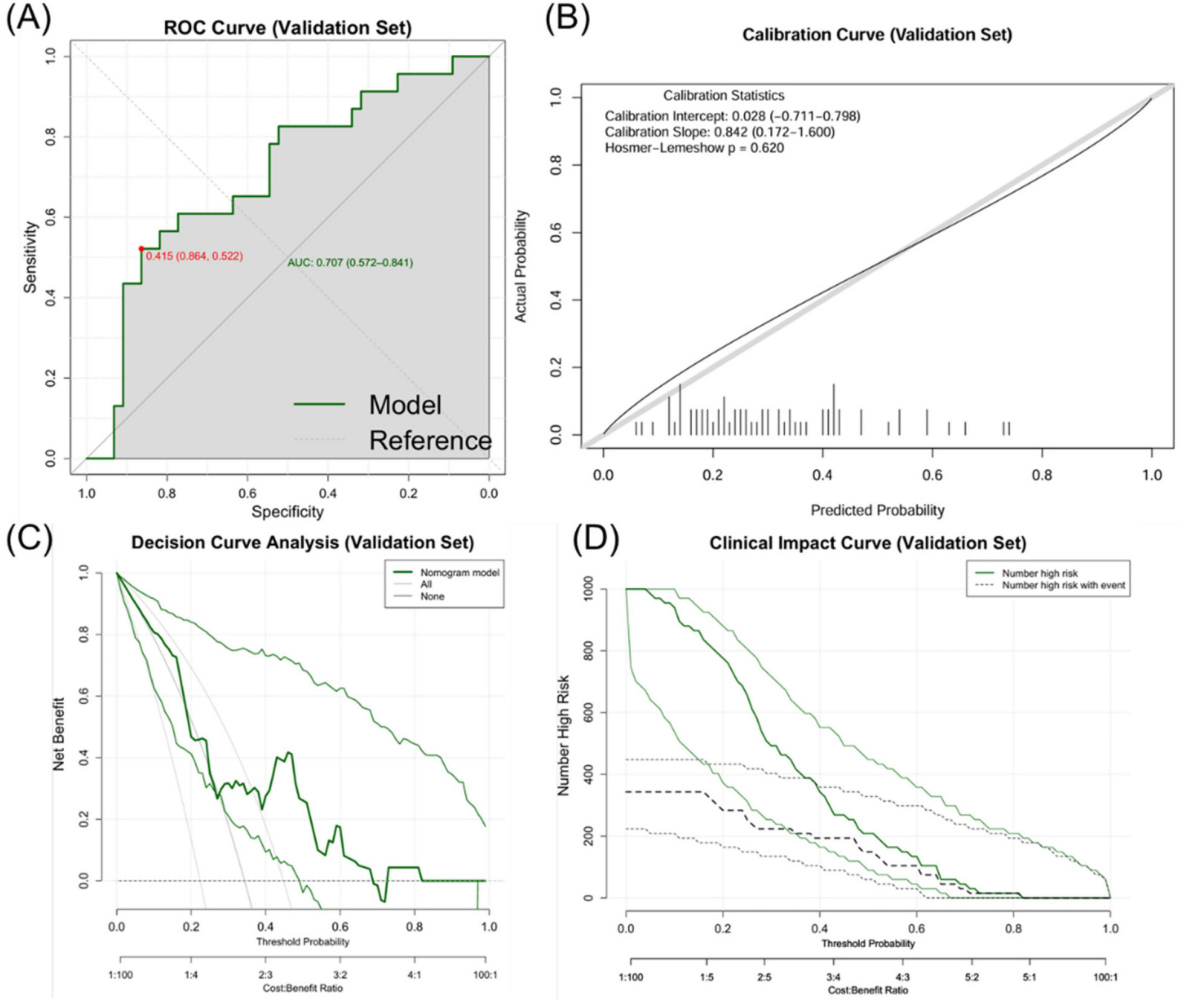
Evaluation of the ISR prediction nomogram (validation set). **(A)** Receiver operating characteristic (ROC) curve. **(B)** Calibration curve. **(C)** Clinical impact curve. **(D)** Decision curve analysis.

The nomogram model exhibited robust performance in both the training and validation sets. In the training set (*n* = 335), the model achieved an original AUC of 0.734 (95% CI: 0.676–0.792) with a bootstrap-corrected AUC of 0.707 after 1,000 resamples, indicating moderate discriminative ability. The Brier score was 0.193 (95% CI: 0.173–0.213), the corresponding calibration slope was 1.000 (95% CI: 0.706–1.359), and the Hosmer-Lemeshow test showed good fit (*χ*^2^ = 8.087, *P* = 0.088) (Table 5). In the independent validation set (*n* = 67), the model exhibited stable performance with an AUC of 0.707 (95% CI: 0.562–0.837), a Brier score of 0.207 (95% CI: 0.161–0.258), a calibration slope of 0.842 (95% CI: 0.229–1.991), and a non-significant Hosmer-Lemeshow statistic (*χ*^2^ = 2.641, *P* = 0.620) (Table 6). DCA revealed clinical utility across 20%–80% threshold probabilities, and clinical impact curves validated net benefit in both cohorts (Figures 5–7).

**Table 5:** Performance metrics of the nomogram (training set).

Performance metric	Original value	Adjusted value	Optimistic value
AUC	0.734	0.707	0.027
Brier Score	0.193	0.204	−0.011

**Table 6:** Overall performance of the ISR prediction nomogram.

Dataset	Number	AUC	AUC confidence interval	Brier score	Brier score confidence interval
Training Set	335	0.734	0.676–0.792	0.193	0.173–0.213
Validation Set	67	0.707	0.562–0.837	0.207	0.161–0.258
Calibration Slope		Calibration Slope Confidence Interval		HL Chi-Square	HL *p*-value
1.000		0.706–1.359		8.087	0.088
0.842		0.229–1.991		2.641	0.620

### 3.7 The predictive value of the substrate material of DES for ISR

The nested model was constructed without incorporating the substrate material of DES. The baseline model (including the substrate material of DES) exhibited an AIC of 403.64, while the reduced model achieved an AIC of 402.16 (ΔAIC = 1.48); however, the likelihood ratio test showed no significant difference (*χ*^2^ = 4.515, *P* = 0.211). In terms of predictive performance, the baseline model yielded an AUC of 0.734 (mean cross-validated AUC: 0.704) and the reduced model (mean cross-validated AUC: 0.706) revealed an AUC of 0.723, with no significant difference (Δ = 0.010, *P* = 0.240). Calibration analysis revealed a good fit for the baseline model (Hosmer-Lemeshow *χ*^2^ = 8.1, *P* = 0.088). On the contrary, the reduced model exhibited significant miscalibration (*χ*^2^ = 14.0, *P* = 0.007) and deterioration of the Brier score from 0.1929 to 0.1962 (Δ = +0.0033). Reclassification metrics showed significant deterioration in continuous NRI (−0.2549, 95% CI: −0.4635 to −0.0481, *P* = 0.021) and IDI (−0.0134, 95% CI: −0.0507 to −0.003, *P* = 0.033), while categorical NRI remained non-significant (−0.0377, *P* = 0.473). DCA confirmed superior net benefit for the baseline model at 10%–70% threshold probabilities. Coefficient stability analysis indicated minimal changes (<5%) in other predictors after excluding the substrate material of DES from the model: BMI (3.7%), SBP (2.9%), LVDD (1.3%), the number of target vessels (3.3%), and the mean diameter of stent (2.2%).

## 4 Discussion

Through a retrospective analysis of 402 patients treated with PCI, this study established and validated a nomogram model incorporating BMI, SBP, LVDD, the number of target vessels, the mean diameter of stent, and the substrate material of DES. The model exhibited stable predictive performance for ISR in both the training set (AUC = 0.734) and the validation set (AUC = 0.707), providing a quantitative tool for clinical decision-making.

### 4.1 Clinical significance of predictive factors

The predictors identified in this model provide an actionable clinical pathway for ISR risk stratification. BMI is a strong modifiable factor, with each 1 kg/m^2^ increase in BMI associated with 13.3% increase in the risk of ISR (OR = 1.133, 95% CI = 1.049–1.224, *P* = 0.002), which is consistent with previous findings (22). Patients with BMI > 30 kg/m^2^ suffer from a higher risk of target lesion revascularization (TLR) (23). Integrating weight management into standard post-PCI care pathways targeting BMI ≤ 25 kg/m^2^ is recommended (24).

It has been reported that the normal BP at the time of PCI is associated with a nearly 24% reduction in the risk of ISR (25). Each 1 mmHg rise in SBP increased the risk of ISR by 1.3% (OR = 1.013, 95% CI = 0.902–1.587, *P* = 0.038) in this study, suggesting that ambulatory blood pressure monitoring can optimize antihypertensive regimens.

Each 1 mm increase in LVDD was associated with 152.5% increase in the risk of ISR (OR = 2.525, 95% CI = 2.455–2.613, *P* = 0.011), indicating superior predictive performance compared to traditional left ventricular ejection fraction (LVEF) (26). High-risk patients (LVDD > 50 mm) should be monitored for the progression of myocardial remodeling through echocardiographic surveillance every 6 months.

Fewer target vessels (OR = 0.762, 95% CI = 0.631–0.921, *P* = 0.005) indicate that more attention should be paid to localized hemodynamic abnormalities in patients with single-vessel disease, necessitating shortened coronary computed tomography angiography (CTA) surveillance intervals (12-month intervals).

The mean diameter of stent is a technically controllable factor where stents ≤2.75 mm increased the risk of ISR by 125% (OR = 0.445, 95% CI = 0.227–0.870, *P* = 0.018), which is consistent with that reported by Lau et al. (27). It was reported that the use of stents ≥3.0 mm is associated with 27% reduction in the risk of restenosis. Therefore, stents with high radial strength (≥3.0 mm) are recommended to reduce target vessel revascularization.

### 4.2 Incremental value and clinical selection of the substrate material of DES

In this model, the substrate material of DES was the only adjustable independent predictor. Removing the substrate material of DES significantly deteriorated the calibration curve (Hosmer-Lemeshow *χ*^2^ = 14.0 vs. 8.1) and reduced the net reclassification index (NRI = −0.2549, IDI = −0.0134). It is widely accepted that calibration quality represents the accuracy of predictions. In clinical practice, physicians rely heavily on the absolute estimates of individual risk factors for decision-making. The inclusion of the substrate material of DES significantly improved the calibration performance of the model. This indicates that the risk prediction model can more accurately reflect the true risk of patients, thereby enhancing its clinical utility. Reclassification metrics (NRI/IDI) focus on the model's ability to correctly reclassify individuals into appropriate risk categories. This precise refinement of risk stratification is crucial for clinical interventions, including preventive measures, surgical planning/stent selection, and postoperative monitoring planning.

Regarding commonly used substrate materials of DES, compared to 316L-SS stents, Co-Cr stents reduced the risk of ISR by 42.9% (Co-Cr vs. 316L-SS: OR = 0.571, 95% CI 0.329–0.992). In complex lesions (bifurcation/calcified), this advantage persisted with a 37% risk reduction, delaying restenosis for two years (28). Co-Cr stents are also suitable for patients with diabetes, small vessel disease, or complex lesions (29), which can be due to their excellent properties, such as thin strut design, high radial strength, and lower nickel ion release (30–32). Pt-Cr stents, despite ultra-thin struts, show no advantage in preventing ISR (OR = 0.702, *P* = 0.431) and are only suitable for simple lesions (33). They are not recommended for bifurcation lesions, chronic total occlusions (CTO) (34) or severe kidney disease (CKD 4–5) (35) due to the risk of longitudinal compression and increased late lumen loss (33, 34). Co-Ni stents can help significantly prevent ISR (OR = 0.671, *P* = 0.341). In addition, the risks of nickel allergy and blood clots should be considered. The nickel allergy patch test may be necessary before application (36). 316L-SS stents show the highest risk of ISR (37) and are only recommended for low-risk patients (vessel diameter ≥ 4.0 mm, 10-year ISR risk <15%). Moreover, the released metal ions (iron, nickel, manganese, chromium) may also induce ongoing inflammation (38).

### 4.3 Clinical application pathway of the predictive model

The constructed nomogram (Figure 5) provides a quantitative tool for managing patients around the time of PCI. The model can be used for preoperative decision support, postoperative monitoring strategies, and integration with healthcare systems. For preoperative decisions, clinicians can use the nomogram's results to evaluate the risks and choose more suitable stents. Co-Cr stents (OR = 0.571) should be preferably used for high-risk patients (score > 150); Pt-Cr stents should not be used for medium-risk patients (score 100–150); and Co-Ni stents may be used for low-risk patients (score < 100) after excluding contraindications. 316L-SS stents are not recommended as the primary device.

After surgery, high-risk patients need anticoagulants, like rivaroxaban, in addition to their dual antiplatelet therapy (DAPT). Furthermore, a coronary angiogram is needed within 3–6 months. Medium-risk patients require more intensive statin treatment. A stress test should be conducted within 6 months and an angiogram is needed within 9 months. Low-risk patients should receive standard 12-month DAPT, followed by coronary CTA after 12 months.

The model can be integrated into the Hospital Information System (HIS), automatically retrieving critical parameters, like LVDD and stent details, to generate real-time risk reports. It can trigger automated alerts for clinicians about high-risk patients and generate detailed follow-up protocols and intervention procedures. DCA confirmed a net benefit of 28 per 100 patients at threshold probabilities between 10%–70%. This can prevent unnecessary angiography and repeat procedures, significantly improving medical resource utilization efficiency.

## 5 Limitations

There are several limitations to this study that should be noted. Firstly, this was a single-center retrospective study conducted at the Affiliated Hospital of Qingdao University. Retrospective designs bring inherent risks of selection bias and information bias. The single-center source may limit the representativeness of the patient population (e.g., specific geographic region, ethnicity, socioeconomic background, and referral patterns), procedural practices (e.g., operator preference, technical details, perioperative management), and selection of drug-eluting stent (DES) types for other centers or broader populations. Furthermore, the model underwent only internal validation using the training/validation sets; therefore, its predictive performance in heterogeneous independent populations remains uncertain. This constrains the generalizability of the nomogram model. Therefore, our findings should be verified in large-scale prospective multicenter cohort studies. Secondly, the exclusion of missing data left a residual sample predominantly composed of highly compliant patients with complete data. These patients exhibited more prototypical disease patterns, which may decrease model performance in the presence of greater heterogeneity.

## 6 Conclusions

A predictive nomogram, including BMI, SBP, LVDD, the number of target vessels, the mean diameter of stent, and the substrate materials of DES, was successfully developed to quantify the risk of ISR. These factors were confirmed as independent predictors of ISR. The model exhibited robust performance with good discriminative ability, calibration consistency, and high clinical utility and was validated through AUC, DCA, CIC, calibration curves, and reclassification metrics (NRI and IDI). This is the first study reporting that the substrate material of DES is a critical factor affecting the risk of ISR.

## Data Availability

The raw data supporting the conclusions of this article will be made available by the authors, without undue reservation.
