# Supplementary material for: A risk prediction nomogram for in-stent restenosis in patients with coronary heart disease: first exploratory analysis based on the substrate materials of drug-eluting stents
Source: Front Cardiovasc Med. 2026 Jan 12;12:1549212. doi: 10.3389/fcvm.2025.1549212 (PMC12832626; doi:10.3389/fcvm.2025.1549212)
Supplement: Supplementary file 1 [file Datasheet1.pdf]

Supplementary materials

S1. Statistical Analysis

S1.1 LASSO regression

The LASSO regression model was fitted using the glmnet package in R, specifying a binomial family distribution and setting alpha = 1 for the standard LASSO penalty. Ten-fold cross-validation was performed to determine the optimal regularization parameter λ. The λ value yielding the minimum cross-validated error (lambda.min) was selected for optimal prediction. Additionally, the largest λ value within one standard error of the minimum cross-validated error (lambda.1se) was noted, which provides a more parsimonious model. Regression coefficients corresponding to lambda.min were extracted. Odds Ratios (OR) were calculated as exp(β).

S1.2 Bootstrap validation for LASSO variable robustness

To validate the stability of variables selected by LASSO, 100 bootstrap resamples with replacement were drawn from the original dataset (n=402). For each bootstrap sample, the entire LASSO modeling procedure using lambda.min identified within that resample was repeated. The frequency (%) with which each candidate variable was selected across the 100 bootstrap samples was recorded. Variables selected in ≥80% of the bootstrap samples were deemed highly stable.

Table S1. Baseline characteristics of the study population.

	Non ISR group (n=258)	ISR group (n=144)	P-value

Age (years old)	61(55,67)	62(56,68)	0.178
Gender, (%)			0.889
Male	188(72.9)	104(72.2)	
Female	70(27.1)	40(27.8)	
Smoking, (%)	99(38.4)	66(45.8)	0.145
Drinking, (%)	73(28.3)	41(28.5)	0.97
BMI,(kg/m^2)	25.655(23.66,27.7)	27.36(24.65,29.905)	<0.001
HR,(beats/min)	66.5(61,76)	67(60,73.75)	0.669
SBP,(mmHg)	130.5(120,147)	136(120.25,150)	0.038
DBP,(mmHg)	76(67,84)	79(70,82.75)	0.073
History of hypertension, (%)	167(64.7)	104(72.2)	0.124
History of diabetes, (%)	104(40.3)	53(36.8)	0.49
History of dyslipidemia, (%)	56(21.7)	37(25.7)	0.363
Family history of CHD, (%)	40(15.5)	24(16.7)	0.76
FBG,(mmo/L)	5.725(4.948,7.413)	5.645(5.03,7.32)	0.555
CHOL,(mmo/L)	4.105(3.37,4.945)	4.26(3.5075,5.03)	0.559
TG,(mmo/L)	1.385(0.948,2.113)	1.32(0.99,1.958)	0.809
HDL,(mmo/L)	1.15(0.998,1.313)	1.09(0.965,1.32)	0.464
LDL,(mmo/L)	2.335(1.83,3.033)	2.52(1.888,3.118)	0.408
GFR, (mL/(min×1.73 m^2))	77.4(67.6,91.55)	78.1(65.96,93.075)	0.92
FIB,(mg/Dl)	2.9(2.425,3.47)	3.03(2.505,3.858)	0.03
LVEF,(%)	62(59,64.25)	60.5(52.25,64)	0.007

LVDD,(mm)	4.6(4.4,4.9)	4.9(4.6,5.2)	<0.001
Form of onset, (%)			0.38
Angina pectoris	157(60.9)	94(65.3)	
Acute myocardial infarction	101(39.1)	50(34.7)	
Number of target lesions	3(2,5)	3(2,4)	0.368
Number of target vessels	3(2,4)	2(2,3)	0.017
Diffuse lesions, (%)	36(14)	15(10.4)	0.307
Degree of stenosis of lesions, (%)	82.5(76.67,88.33)	82.5(76.67,90)	0.455
Number of implanted stents	2(1,3)	2(1,3)	0.367
Mean diameter of stent,(mm)	2.88(2.63,3.08)	2.75(2.56,3)	0.029
Mean length of stent,(mm)	22(18,26.543)	23(18.25,28)	0.024
Minimum diameter of stent,(mm)	2.75(2.5,3)	2.5(2.5,2.75)	0.071
Target vessel,(%)			
left main artery	9(3.5)	7(4.9)	0.5
Intermediate branch	6(2.3)	10(6.9)	0.023
First diagonal branch	57(22.1)	45(31.3)	0.106
Second diagonal branch	18(7)	12(8.3)	0.113
Proximal left anterior descending artery	137(53.1)	82(56.9)	0.458
Middle left anterior descending artery	128(49.6)	58(40.3)	0.072
Distal left anterior descending artery	30(11.6)	19(13.2)	0.645
Proximal left circumflex	61(23.6)	40(27.8)	0.359
Middle left circumflex	93(36)	55(38.2)	0.669

Distal left circumflex	57(22.1)	27(18.8)	0.429
First blunt marginal branch	35(13.6)	23(16)	0.052
Second blunt marginal branch	6(2.3)	3(2.1)	0.875
Proximal right coronary artery	74(28.7)	43(29.9)	0.803
Middle right coronary artery	96(37.2)	46(31.9)	0.29
Distal right coronary artery	60(23.3)	27(18.8)	0.293
Left ventricular posterior branch	23(8.9)	18(12.5)	0.222
Right ventricular posterior branch	1(0.4)	1(0.7)	0.675
Posterior ventricular branch	39(15.1)	13(9)	0.081
Substrate material of DES,(%)			0.046
316L-SS	86(33.3)	65(45.1)	
Co-Cr	118(45.7)	46(31.9)	
Pt-Cr	28(10.9)	16(11.1)	
Co-Ni	26(10.1)	17(11.8)	
